# Disability in activities of daily living, depression, and quality of life among older medical ICU survivors: a prospective cohort study

**DOI:** 10.1186/1477-7525-9-9

**Published:** 2011-02-05

**Authors:** Michael T Vest, Terrence E Murphy, Katy LB Araujo, Margaret A Pisani

**Affiliations:** 1 Section of Pulmonary and Critical Care Medicine, Department of Medicine, Yale University School of Medicine, 333 Cedar Street, PO Box 208057, New Haven, CT 06520-8057 USA; 2 Section of Geriatrics, Department of Internal Medicine, Program on Aging, Yale University School of Medicine, 333 Cedar Street, PO Box 208057, New Haven, CT 06520-8057 USA; 3 Section of Pulmonary and Critical Care Medicine, Department of Medicine, Program on Aging, Yale University School of Medicine, 333 Cedar Street, PO Box 208057, New Haven, CT 06520-8057 USA

## Abstract

**Background:**

Accurate measurement of quality of life in older ICU survivors is difficult but critical for understanding the long-term impact of our treatments. Activities of daily living (ADLs) are important components of functional status and more easily measured than quality of life (QOL). We sought to determine the cross-sectional associations between disability in ADLs and QOL as measured by version one of the Short Form 12-item Health Survey (SF-12) at both one month and one year post-ICU discharge.

**Methods:**

Data was prospectively collected on 309 patients over age 60 admitted to the Yale-New Haven Hospital Medical ICU between 2002 and 2004. Among survivors an assessment of ADL's and QOL was performed at one month and one-year post-ICU discharge. The SF-12 was scored using the version one norm based scoring with 1990 population norms. Multivariable regression was used to adjust the association between ADLs and QOL for important covariates.

**Results:**

Our analysis of SF-12 data from 110 patients at one month post-ICU discharge showed that depression and ADL disability were associated with decreased QOL. Our model accounted for 17% of variability in SF12 physical scores (PCS) and 20% of variability in SF12 mental scores (MCS). The mean PCS of 37 was significantly lower than the population mean whereas the mean MCS score of 51 was similar to the population mean. At one year mean PCS scores improved and ADL disability was no longer significantly associated with QOL. Mortality was 17% (53 patients) at ICU discharge, 26% (79 patients) at hospital discharge, 33% (105 patients) at one month post ICU admission, and was 45% (138 patients) at one year post ICU discharge.

**Conclusions:**

In our population of older ICU survivors, disability in ADLs was associated with reduced QOL as measured by the SF-12 at one month but not at one year. Although better markers of QOL in ICU survivors are needed, ADLs are a readily observable outcome. In the meantime, clinicians must try to offer realistic estimates of prognosis based on available data and resources are needed to assist ICU survivors with impaired ADLs who wish to maintain their independence. More aggressive diagnosis and treatment of depression in this population should also be explored as an intervention to improve quality of life.

## Background

Physicians and patients face difficult choices when deciding goals of care in the face of critical illness. We often look to the medical literature for data to help us guide our patients and their families. Traditionally, the critical care literature has been focused on mortality, which has been described as a "hard outcome" with implication that it is more valid than other "soft outcomes". Secondary or physiologic outcomes are also commonly chosen for intensive care unit (ICU) research. A major limitation of these outcomes is their relevance to patient function after discharge.

Mortality in critically ill patients is impacted by severity of illness, comorbidities, and, pre-morbid functional status. Importantly, the decision not to provide life support has been shown to predict mortality independent of comorbidities and severity of illness [1]. While these factors result in significant variability in mortality based on population studied, mortality in critically ill older patients is universally high. For example, in analysis of 65-74 year old patients mortality by hospital discharge was 40% [2], in a cohort of patients over age 70 with long ICU stays, mortality at hospital discharge was 53% [3] and in a recent study of patients over age 80 mortality at hospital discharge was 45% [4]. However, many patients would be willing to accept a high risk of death, if the potential reward is a high quality of life.

Quality of life (QOL) is an important outcome because it is patient centered and clinically meaningful. Health related quality of life (HRQOL) is that portion of quality of life determined by one's health. HRQOL is made up of physical, psychological, and social domains which interact with each other and with the patient's perceptions[5]. From here on in this paper, all references to quality of life refer to health related quality of life.

The literature on quality of life in ICU survivors is mixed. A recent review summarized numerous studies documenting severe cognitive decline, psychiatric illness, and impaired quality of life in survivors of critical illness[6]. For example, an analysis of Acute Respiratory Distress Syndrome survivors showed that these patients had a lower quality of life as long as 66 months after ICU discharge [7]. However, in reviewing a cohort of 115 patients greater than age 80 who received ICU care in France, the 23 patients who survived to one year follow-up not only had quality of life similar to age and sex matched controls but also experienced no decline in functional status compared to before their ICU care[4]. Further, Montuclard et al reported that among the subset of a French cohort of elderly patients who received prolonged ICU stays (>30 days) and survived, quality of life was sufficient to recommend aggressive ICU treatment[3]. The results from the French cohort contrast with the poor outcomes (9% alive and independent at one year) reported in a US population of adult patients receiving prolonged mechanical ventilation[8]. However, there is evidence that well planned interventions, such as early initiation of physical therapy or therapeutic hypothermia after cardiac arrest, may improve quality of life in survivors of critical illness [9,10].

Measuring quality of life in survivors of ICU admission is complicated by the fact that many of these patients may be unable to answer questions required for use of validated quality of life measures, such as the SF-12. This is particularly true of geriatric survivors. Thus, the investigator is left with the question of how to measure quality of life in these patients. For example, can QOL be accurately gauged from responses of surrogates or care givers?

QOL measurements are further complicated by the fact that QOL is not static and thus, the timing of when QOL is assessed may greatly impact the results[6]. Several studies including work with survivors of acute lung injury suggest that QOL may improve over the first six months after ICU discharge [6,11]. However, the optimal timing of QOL measurement is not known, especially in older populations with high short term mortality.

Andersen et al correlated quality of life with disability in activities of daily living (ADLs) [12]. However, this relationship has not been specifically addressed in survivors of critical illness. They found the inability to independently perform ADLs was the major factor affecting quality of life. Since the ability to independently perform ADLs can be objectively observed by a proxy or investigator, it is an appealing marker for quality of life. Additionally, in older patients who survive an ICU stay, it seems intuitive that the physical domain (partially measured by ADL independence) would have a large impact on other domains of quality of life. Therefore, we decided to investigate the cross-sectional associations between disability in ADLs and quality of life (SF-12) at one month and one year post-ICU discharge in a cohort of older medical ICU survivors.

## Methods

**Figure 1:**
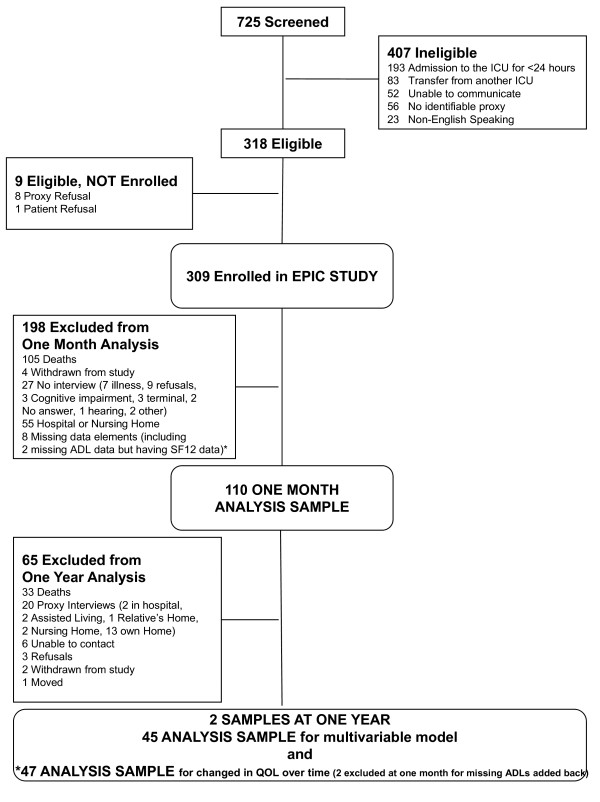
**Screening and Eligibility Flow Diagram**.

Our cohort consisted of 309 consecutive patients 60 years or older who were admitted to the medical ICU at Yale-New Haven Hospital, New Haven, Connecticut, from September 5, 2002 through September 30, 2004. Yale-New Haven hospital is a large teaching hospital with a 28-bed medical ICU. The decision to admit a patient to the ICU was at the discretion of the attending physician. Data was collected after study approval by the institutional review board. Patients were excluded if no proxy was available to provide information, they died before the proxy interview was obtained, they were transferred from another ICU, their admission lasted less than 24 hours or they were non-English speaking. All medical ICU admissions of patients age 60 and over during this time period were screened for enrollment. Figure 1 shows the screening and enrollment process. Of this cohort, analysis was restricted to the patients with quality of life and other co-variables available at one month and one year post-ICU discharge.

Data was collected by trained research nurses. Standardization included inter-rater reliability assessments for all key measures. ICU admission data included patient demographics and the Acute Physiology and Chronic Health Evaluation II (APACHE II) score. Screening for pre-existing dementia was based on interviews conducted with surrogates, upon patient enrollment into the study, using the Informant Questionnaire on Cognitive Decline in the Elderly (IQCODE)[13]. The patients were followed throughout their hospitalization and interviewed one month and one year after ICU discharge. The one month and one year post-discharge interviews were conducted via telephone by trained nurses using scripted text with both patients and surrogates. ADLs were assessed using Katz's ADL measures and quality of life measured by SF-12 [14]. Due to concerns about reliability, surrogates were not allowed to answer SF-12 questions. Thus, all quality of life data was obtained directly from patients. Physical and mental composite scores were calculated according to SF-12 scoring guidelines for version one norm based scoring standardized to 1990 population norms (i.e., the mean score of 50 points represents the mean for the general US population) [15]. Additionally, the interviewed patients were screened for depression using a two question screening tool[16], for delirium using the Confusion Assessment Method-ICU [17], and for use of health care services since discharge. The 2 question depression screening tool was developed for use in primary care and can easily been administered during an interview. It has been reported to have a sensitivity of 96% and a specificity of 57%[16].

### Statistical Analysis

Descriptive statistics were ascertained as appropriate. Because the outcomes (SF-12 physical and mental summaries) were normally distributed, we used multiple linear regression. Our main predictor was any impairment in ADL. ADL scores were skewed; and, thus, were handled as a dichotomous variable: any impairment versus completely independent. For adjustment purposes, control variables were selected a priori on clinical grounds and forced into the multivariable model. These included age, race, gender, education, Charlson Comorbidity Index score [18], intubation during ICU stay, length of ICU stay, depression, total days of delirium, and APACHE II score [19].

As depicted in Figure 1, our analytical sample was a fraction of the original cohort and subject to several causes of missingness not plausibly assumed to be missing at random. For this reason no imputation was performed. Model fit was assessed with residual analysis. A p-value of 0.05 was considered to be significant for all two-sided statistical tests. Among the subgroup that survived through one year post ICU discharge, we performed supplementary analysis examining differences in SF-12 scores from one month to one year and created a regression model to examine the cross-sectional association between ADLs and QOL at one year. Due to mortality related reduction in power at one year, control variables in this model were limited to age, gender, race and the Charlson Comorbidity Index Score. A paired t-test was used to determine if SF-12 scores at one year were different from those at one month. A Spearman correlation was performed to examine the association between ADLs and depression. SAS statistical software, version 9.2 (SAS Institute Inc., Cary, North Carolina), was used for all analysis [20].

## Results

Of the 309 patients enrolled in the cohort, 110 had all data required for regression models available at one month post-ICU discharge. Figure 1 presents our enrollment process. Of 199 patients not included in model at one month post-ICU discharge, 105 were deceased, 24 were hospitalized 31 were in a nursing home, 27 were not interviewed (10 due to illness--including 3 terminally ill, 9 due to refusals, 3 due to cognitive impairment, 2 could not be contacted, 1 due to hearing impairment and 2 for other reasons), 8 were missing data, and 4 withdrew from the study. Table 1 presents demographic data on our patient population. The average age was 72.6 ± 8.3 years, with 45% being male and 89% admitted to the ICU from home. At ICU admission persons with the post-discharge QOL data were significantly younger (mean age 72.6 v. 75.9), had lower APACHE II scores (mean 21.4 v. 24.6), were more likely to have been admitted from home and were less likely to have a positive screen for pre-existing dementia or depression. At ICU admission this subset was also significantly less likely to need help with activities of daily living than patients without QOL data (18% v. 46% with p < 0.0001).

**Table 1:** ICU Admission Characteristics of Patients in Full Cohort, Excluded Patients, and One Month Analysis Sample

Characteristic	Full Cohort **(n = 309) **†	Excluded **(n = 199) **†	One Month Analysis Sample **(n = 110) **‡	**P-value**††
	Mean (SD) or n (%)	Mean (SD) or n (%)	Mean (SD) or n (%)	
Age in years	74.7 (8.5)	75.9 (8.3)	72.5 (8.3)	0.001
Male	145 (47)	96 (48)	49 (45)	0.53
Education in years	12.5 (2.8)	12.4 (2.9)	12.5 (2.6)	0.75
Non-white race	51 (16)	35 (18)	16 (14)	0.49
APACHE II score	23.5 (6.4)	24.6 (6.3)	21.4 (6.1)	<0.0001
Charlson Co-Morbidity Index	1.8 (1.9)	1.9 (2.0)	1.7 (1.6)	0.99
Admitted from home*	241 (78)	143 (72)	98 (89)	0.0005
**Baseline Medical Status**				
				
Evidence of depression**	85 (28)	63 (32)	22 (20)	0.03
Dementia	95 (31)	75 (38)	20 (18)	0.0004
Any Impairment in Activities of Daily Living	115 (37)	94 (47)	21 (19)	<0.0001
Bathing impairment	104 (34)	88 (44)	16 (14)	<0.0001
Grooming impairment	42 (14)	37 (19)	5 (5)	0.0005
Transfer bed to chair impairment	54 (17)	48 (24)	6 (5)	<0.0001
Walk across room impairment	56 (18)	49 (25)	7 (7)	<0.0001
Ability to dress impairment	44 (14)	34 (17)	10 (9)	0.05
Ability to eat impairment	10 (3)	10 (5)	0 (0)	0.02
Ability to toilet impairment	41 (13)	38 (19)	3 (3)	<0.0001
**Admitting Diagnosis**				
				
Sepsis	51 (16)	39 (20)	12 (11)	0.049
Respiratory	156 (51)	104 (52)	52 (47)	0.40
Neurologic	5 (2)	5 (3)	0 (0)	0.16
Gastrointestinal hemorrhage	52 (17)	24 (12)	28 (25)	0.003
Other	45 (15)	27 (14)	18 (16)	0.50
**ICU Factors**				
				
Delirium during ICU Stay***	239 (79)	177 (92)	61 (55)	<0.0001
Intubated	167 (54)	126 (63)	41 (37)	<0.0001
Days of ventilation, Median (IQR)****	6 (8)	6 (10)	4 (3)	0.006
				
Length of stay, Median (IQR)****	5 (6)	6 (9)	3 (3)	<0.0001
				
Total days of delirium (IQR)****	5 (6)	6 (9)	1 (3)	<0.0001

†Missing data present for some subjects. For Dementia missing = 3; Charlson Co-Morbidity missing = 1; Education missing = 9; Delirium missing = 5

† Missing data present for some subjects. For Dementia missing = 1; Grooming impairment missing = 2; Ability to dress impairment missing = 2; Ability to eat impairment missing = 1; Ability to toilet impairment missing = 2

‡Missing data present for some subjects. For Dementia missing = 1;

*Admitted from home versus Skilled Nursing Facility or Rehabilitation Center

**Evidence by surrogate or chart

***Delirium by CAM interview or chart review during entire ICU stay

****During entire ICU stay (includes first admission and, if applicable re-admissions to ICU)

††Comparison of excluded (n = 199) and analysis sample (n = 110): Chi-square or Fisher's Exact for categorical variables and T-test or Wilcoxon test as appropriate for continuous variables

In our full cohort of 309 older patients, mortality was 17% (53 patients) at ICU discharge, 26% (79 patients) at hospital discharge, 33% (105 patients) at one month post ICU admission, and 45% (138 patients) at one year. Moreover, for our total cohort 52% of participants were either deceased or living in institutions at one-month post ICU discharge.

The physical component SF12 scores averaged 31 which is significantly below the population mean of 50 ± 10. The mental component score of the SF-12 averaged 51, which is not significantly different than population mean of 50 ± 10. Table 2 presents the results of our multivariable regression models for SF-12 PCS and MCS at one month. After adjusting for clinically important covariates in the PCS model, ADL disability at one month was associated with significantly worse quality of life (β = -7.11; p < 0.0001) as was depression (β = -3.62; p = 0.03. In the MCS model, only depression showed a significant association (β = -8.71; p < 0.0001), ADL disability was not statistically significant. As can be seen in both columns of Table 2, age, race, gender, education, comorbidities, ICU length of stay, intubation, days of delirium, and APACHE II score were not significantly associated with either PCS or MCS scores at one month post-ICU discharge.

**Table 2:** Multivariable Model Results for SF12 Physical and Mental Component Scores Measured One Month Post-ICU Discharge (N = 110)*

Explanatory Variables	Physical Component (PCS)		Mental Component (MCS)	
**Explanatory Variables**	**β (95% CI)**	**P-value**	**β (95% CI)**	**P-value**

Any Impairment in Activities of Daily Living (ADL)	-7.11 (-10.43, -3.80)	<0.0001	-3.02 (-6.59, 0.55)	0.10
ICU Length of Stay	-0.40 (-1.43, 0.63)	0.44	-0.40 (-0.70, 1.51)	0.47
Intubation	0.50 (-4.60, 3.60)	0.81	0.82 (-3.59, 5.23)	0.71
Age	0.07 (-0.12, 0.27)	0.49	0.10 (-0.11, 0.32)	0.35
APACHE II Score on ICU admission	-0.06 (-0.36, 0.23)	0.67	-0.05 (-0.26, 0.36)	0.74
Charlson Co-morbidity Index	-0.44 (-1.41, 0.55)	0.37	0.72 (-0.33, 1.76)	0.18
Education	-0.20 (-0.81, 0.42)	0.53	-0.03 (-0.69, 0.63)	0.93
Male Gender	0.27 (-2.92, 3.46)	0.87	0.79 (-2.64, 4.23)	0.64
Nonwhite Race	-0.44 (-5.04, 4.15)	0.85	-1.28 (-6.23, 3.66)	0.61

Depression	-3.62 (-6.86, -0.38)	0.03	-8.71 (-12.20, -5.22)	<0.0001

Days of Delirium	0.51 (-0.53, 1.56)	0.33	-0.47 (-1.60, 0.65)	0.40

* Abbreviations: CI, Confidence Interval; ICU, Intensive Care Unit; APACHE, Acute Physiology and Chronic Health Evaluation

Age, APACHE II Score, Charlson Co-morbidity Index and Education are all continuous variables. ADLs were measured at 1-month post-ICU discharge.

R^2 ^= 0.26 for Physical Component and R^2 ^= 0.28 for Mental Component

Adjusted R^2 ^= 0.17 for Physical Component and Adjusted R^2 ^= 0.20 for Mental Component

Our multivariable model of PCS explained 17% of the variability in SF-12 PCS; while our model of MCS explained 20% of the variability. Both depression and ADL dependence were statistically significant variables in the PCS model but only depression reached statistical significance in the MCS model. Depression was correlated with ADL impairment with a coefficient of -0.20 (p = 0.04).

As shown in Table 3, there was a high prevalence of impairment of ADLs in this cohort. Bathing impairment was seen in 62% of the cohort at one month. Those who survived to one year continued to have frequent bathing impairment (36%). Table 4 shows ADL impairment and mean quality of life scores at one month and one year.

**Table 3:** Activities of Daily Living at One Month and One Year Follow-up Interview in Full Cohort and Analysis Sample

Characteristic	All Subjects with ADL data		Analysis Sample	
	**One Month** (n = 200)*	**One Year** **(n = 103)**	**One Month** **(n = 110)**	**One Year** **(68/110)**
	**n (%)**		**n (%)**	

Impairment in Activities of Daily Living **				
Bathing	123 (62)	37 (36)	43 (39)	14 (21)
Grooming	81 (41)	11 (11)	18 (16)	3 (4)
Transfer bed to chair	80 (40)	14 (14)	17 (15)	5 (7)
Walk across room	82 (41)	16 (16)	18 (16)	5 (7)
Ability to dress	87 (44)	23 (22)	19 (17)	7 (10)
Ability to eat	53 (27)	8 (8)	6 (5)	4 (6)
Ability to toilet	75 (38)	12 (12)	11 (10)	4 (6)

*Full Cohort minus 105 patients who died prior to one month follow-up and 4 patients without data on ADLs due to withdrawal from study

**Impairment defined as requiring help or unable to do activity as reported by patient or surrogate (at One year 20 surrogates provided information for patients who could not be interviewed)

***Column numbers do not add up to total number of patients because some patients have impairment in more than one ADL and other patients do not have impairment in any ADLs

**Table 4:** SF12 Physical and Mental Component Scores and Activities of Daily Living at One Month and One Year Follow-up Interview

Characteristic	One Month	One Year
Any Impairment in Activities of Daily Living *	47 (42%)	14 (21%)
SF12 Physical Component Score**	37.2 (8.7)	43.6 (10.7)
SF12 Mental Component Score **	51.5 (9.5)	54.9 (7.3)

*Impairment defined as requiring help or unable to do activity as reported by patient or surrogate on any of 7 Basic Activities of Daily Living (at One year 20 surrogates provided information for patients who could not be interviewed). ADL data on 111 patients at one month and 68 patients at one year. Due to patients missing other data elements this is more patients than could be included in models.

**Quality of Life Data is shown for 111 patients at one month and 45 patients at one year. It does not include 2 patients with QOL data at one month and one year exclude from analysis. Data is presented as mean SF-12 score with standard deviation in parentheses.

**Figure 2:**
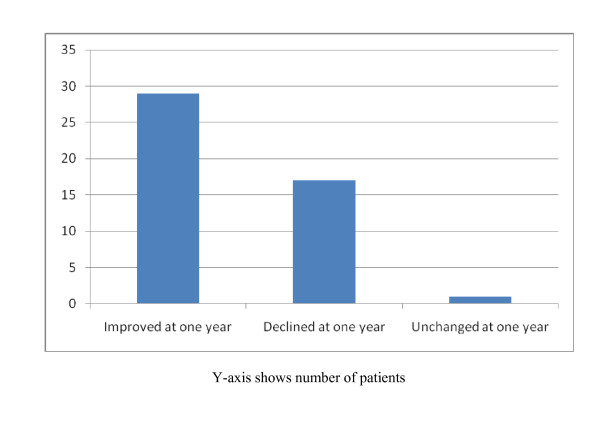
**Comparison of SF-12 Physical Component Scores from One Month and One Year**.

There were 47 patients from the total cohort who survived in the community and had QOL data collected at both one month and one year. For this subset of patients the mean SF-12 MCS at one month and one year were 53 and 55, respectively. Changes in SF-12 MCS scores between one month and one year were not statistically significant (p = 0.17). The mean PCS at one month was 39 but increased to 43 at one year, representing a significant change in PCS scores over time (p = 0.014). On average this change was an improvement. However, as shown in Figure 2, 17 patients (36%) actually experienced a reduction in quality of life as measured by SF-12 PCS score, 29 (61%) saw an improvement in QOL as measured by PCS score, and one patient (2%) had no change in PCS score.

Of these 47 patients, two were missing data on ADLs and thus could not be included in regression analysis. An analysis of the remaining 45 patients shown in table 5 revealed that ADL dependence at one year was not associated with either PCS or MCS scores. Moreover, neither were any of the covariates of age, charlson comorbidity index, race or gender statistically significant.

**Table 5:** Multivariable Model Results for SF12 Physical and Mental Component Scores Measured One Year Post-ICU Discharge (N = 45)*

Explanatory Variables	Physical Component		Mental Component	
**Explanatory Variables**	**β (95% CI)**	**P-value**	**β (95% CI)**	**P-value**

Any Impairment in Activities of Daily Living (ADL)	-10.71 (-25.69, 3.25)	0.13	7.02 (-2.96, 16.99)	0.16
Age	-0.32 (-0.75, 0.11)	0.15	0.06 (-0.25, 0.37)	0.70
Charlson Co-morbidity Index	-1.21 (-3.26, 0.83)	0.24	0.89 (-0.57, 2.35)	0.22
Male Gender	2.24 (-4.48, 8.99)	0.89	1.16 (-2.63, 4.96)	0.54
Nonwhite Race	-5.55 (-15.07, 3.9)	0.50	0.57 (-4.24, 5.38)	0.81

R^2 ^= 0.19 for Physical Component and R^2 ^= 0.11 for Mental Component

Adjusted R^2 ^= 0.09 for Physical Component and Adjusted R^2 ^= 0.0.01 for Mental Component

## Discussion

In this study we describe QOL outcomes at one month post-ICU discharge in a cohort of older survivors of a medical ICU admission. We hypothesized that disability in ADLs might explain much of the quality of life achieved or lost in this population shortly after life threatening physical illness. Our one month model explains 17% of the variance in the PCS and 20% of the variance in the MCS. The impact of ADL disability is consistent with the findings of Andersen et al [12], who found a correlation coefficient of 0.289 for ADL independence and quality of life. In contrast, our one year model did not reveal an association with functional status and quality of life. This may be due to the absence of an association or due to loss of power due to small number of patients.

The impact of depression on both PCS and MCS is a clinically important finding. Depression is known to occur in 25 to 50% of critical illness survivors[6]. There are many studies analyzing the incidence and risk factors for mental illness (both depression and post-traumatic stress disorder); however, it may be time for trials of aggressive case finding and intervention. As safe, highly effective therapies are available for depression, more aggressive diagnosis and treatment may be indicated to improve quality of life in this population.

Intuitively one might expect that poor physical health would result in poor mental health with corresponding decline in QOL. The reasons that this was not observed in our study are not clear. Perhaps these patients felt that they were getting better physically and had high hopes for future improvement. This hypothesis would be consistent with reports that QOL improves during serial follow-up after ICU discharge [7]. It is also possible that this group of older patients has a higher tolerance for physical problems. We did observe negative associations between depression and SF-12 scores (PCS and MCS), and a negative correlation between depression and ADL independence. So, poor mental health appears to have a significant impact on physical health in this population.

Approximately 50% of the observed mortality in our cohort occurred after discharge from the ICU. The hospital mortality for this group of older patients was higher than the 13.8% described by Higgins et al in 2007 and similar to the mortality of 39% reported by Chelluri et al in 1993 for older ICU patients [2,21]. Additionally, the in-hospital mortality was equivalent to that reported by Pisani et al in a separate cohort of 395 patients [22]. Our cohort had slightly lower mortality than the cohorts reported by Tabah and Boumedil; however, our patients were on average younger [4,23]. Despite the high mortality (45%) and low incidence of independent living at one year, the 15% of the cohort who survived and were community dwelling at one year had a relatively good QOL (mean PCS-43, mean MCS-55). This is similar to the findings of Tabah et al who reported a high one year mortality (68.9%) but good quality of life among the subset of octogenarians who survived ICU care and lived to one year follow-up[4]. We identified the subset of patients from a large cohort of older patient admitted to a tertiary care ICU with the best outcomes. This higher performing subset was less likely to have been cognitively impaired or dependent with regard to ADLs prior to ICU admission. This is consistent with prior findings that poor functional status prior to ICU admission portends a poor prognosis. The differences between our subset and the larger cohort including age, diagnosis, APACHE II score, and pre-morbid health status deserve further investigation as possible prognostic factors for older patients admitted to the ICU.

One option for improving independence suggested by this work is optimizing community support for ADLs such as bathing. We found 39% of our high performing subset and 62% of survivors overall were unable to bath themselves one month after ICU discharge. Discharge planning addressing this need may allow some currently institutionalized survivors to return to community living. Research needs to be done to find ways of improving independence after ICU discharge in older patients and to help inform patients and families of expected outcomes.

This study has several limitations: first, we analyzed a prospectively collected dataset for which quality of life was not the primary outcome. Although version two of the SF-12 was available at the time of data collection, the older version was used. We do not have access to the more recent population norms or the 1990 normative data that would allow comparison with age and sex matched controls. Although it would be optimal to have more recent norms matched by age and sex, the associations noted between ADL impairment and QOL, and between depression and QOL hold true regardless of the whether population norms or age and sex matched controls are used.

Data from validated quality of life measures was only available for cognitively intact community dwelling survivors healthy enough to answer SF-12 survey questions for themselves. While this limits the generalizability of our findings, it also serves to emphasize one of the problems that inspired this study: how to measure quality of life in the population of survivors who cannot respond to a validated quality of life survey tool. Both quality of life and functional status can change with time. Prior studies as well as our own data from the small subset of patients for whom we have SF-12 data at both one month and one year suggest that quality of life may improve with time. The optimal time to measure outcomes has yet to be determined and, in fact, a single point in time measurement may be inadequate. However, in a population with a 33% one month mortality, we feel that short term outcomes are important.

We describe QOL outcomes in a large cohort of older ICU patients. The size of this cohort compares favorably to other studies of QOL in older ICU patients such as the 97 patients reported on by Chelluri et al and 180 reported by Garrouste-Orgeas et al [2,24]. Moreover, our use of a rigorously validated QOL measure and data collection via structured interviews by trained research nurses ensure a high degree of internal validity to this data.

Data suggests that critical care physicians in the United States need to do better at communicating QOL expectations to patients and their families[8]. Cohorts such as ours can help inform our thinking on outcomes in older patients and in the future, perhaps, help us identify patients most likely to benefit from intensive care. In the short term; however, our findings suggest that discharge planning incorporating support for ADLs such as bathing and aggressive screening and treatment for depression might improve quality of life in this population.

Further research directed at developing and validating QOL tools better suited to ICU survivors is needed. The ideal tool would allow stratification of QOL states based on objective observations of patients unable to participate in surveys or interviews. Alternatively, further validation of QOL measurement based on surrogate responses would be welcomed. However, in the absence of a gold standard for use in the ICU, investigators should continue to use validated QOL measures, such as the SF-12, SF-36 and EuroQol, to determine QOL in various patient populations.

## Conclusions

Survivors of critical illness have reduced quality of life especially in the physical domains. Functional status as measured by ADL disability and depression are the best predictors of quality of life in multivariable analysis. Our model explained 17% of variability in physical component quality of life scores and 20% of variability in mental component scores at one month. This degree of correlation is not adequate to allow functional status to serve as the sole surrogate marker for quality of life. Discharge planning for ICU survivors should incorporate both support for ADLs such as bathing and aggressive screening and treatment of depression.

## Abbreviations

QOL: quality of life; SF-12: short form 12-item health survey; SF-36: short form 36 item health survey; ADLs: activities of daily living; PCS: physical component score; MCS: mental component score; ICU: intensive care unit; APACHE II: Acute Physiology and Chronic Health Evaluation II; HRQOL: Health related Quality of Life; IQCODE: Informant Questionnaire on Cognitive Decline in the Elderly;

## Competing interests

The authors declare that they have no competing interests.

## Authors' contributions

MP designed cohort study. All authors participated in data analysis. MTV developed research question and drafted manuscript which has been approved by all authors. MP and KA supervised data collection. TM performed or supervised all statistical analysis.
